# Experimental study on fire characteristics in cable compartment of utility tunnel with natural ventilation

**DOI:** 10.1371/journal.pone.0266773

**Published:** 2022-04-08

**Authors:** Z. P. Bai, H. W. Yao, H. H. Zhang

**Affiliations:** 1 Department of College of Building Environment Engineering, Zhengzhou University of Light Industry, Zhengzhou, China; 2 Zhengzhou Real Estate Group Investment Management Co., Ltd, Zhengzhou, China; University of Genova, ITALY

## Abstract

The fire characteristics under natural ventilation in the cable compartment of the utility tunnel are studied. A series of small-scale fire experimental tests are conducted to obtain the maximum temperature below the ceiling of different ignition vertical heights and cable types. In this paper, the ceiling temperature decay and heat release rate (HRR) are studied in the cable compartment of utility tunnel. Through experimental tests, the fire characteristics of placing the fire source on the near wall side 3.0 m away from the shaft of the utility tunnel cable compartment are studied. The results showed that under the action of natural ventilation, with the decrease of fuel quantity, the mass loss of cable decreases, and the maximum temperature below the ceiling of the cable compartment in the utility tunnel decreases. ZRYJV cables burn more sufficient combustion than RVVR cables. A new empirical association for total HRR is proposed. Those experimental test results are used as validation data for the newly proposed empirical correlation of total HRR. This paper hopes to provide some basic fire safety references for the utility tunnel planning of the urban underground cable compartment.

## 1. Introduction

With the rapid development of social production demand and the increase of urban population, the fire in cable compartment is becoming more and more serious. The continuous application and further development of urban underground space has become an inevitable trend. In case of fire in the utility tunnel, it is very dangerous [1]. In case of fire in the cable compartment, the smoke is difficult to spread vertically, and can only be discharged from the vents at both ends [2]. Therefore, smoke tends to propagate along the longitudinal direction of the cable compartment. Usually, a small fire in a cable compartment can lead to a huge disaster [3]. In case of fire, the temperature will change with the change of ventilation velocity and heat release rate (HRR), and the temperature distribution will also change with the passage of time [4].

Previous studies mostly used numerical simulation methods to study the characteristics of tunnel fire [5]. Zhao [6] proposed a simplified model of corridor ceiling jet behavior. The model is verified by numerical calculation. For the radial expansion zone, the Alpert equation is simplified and the simplified solution is obtained. Ji [7] studied the influence of tunnel aspect ratio on smoke temperature distribution near the ceiling of the fire source area. The results showed that the tunnel width had little influence on the smoke mass loss rate and the maximum smoke temperature, but had a great influence on the smoke temperature distribution. Vaquelin and Wu [8] and van Maele and Merci [9] found that the critical ventilation velocity was related to the tunnel width of Computational Fluid Dynamics (CFD). The Fire Dynamics Simulator (FDS) solved the problems of smoke propagation and heat transfer in fire [10]. However, for the simulation of cable compartment, a large number of grids need to be established for long-time simulation. The length of the near fire source region is a key factor in the simulation method. However, it is not commonly used in the numerical simulation of smoke propagation in cable compartment.

In the past few years, a series of fire tests in cable tunnels have increased significantly after deadly tunnel fires in Europe. Unlike small-scale tests [11] and large-scale tests (such as those conducted in Zwenberg (Austria), Ofenegg(Switzerland), or Finland [12]), the Memorial tunnel experimental test showed that it was consistent with the experimental measurement results in the far field of the fire source [13]. However, for the length of near fire source area, the calculation is not accurate. Fan [14] conducted experimental research in a 1:20 model tunnel, studied the flame length integral model considering the influence of tunnel section and ventilation velocity under large HRR near the fire source, and proposed a dimensionless ventilation velocity form considering the hydraulic diameter of the tunnel. Through a series of fire experiments, Jiang [15] studied the phenomenon of the central smoke exhaust system of the tunnel through a series of fire experiments, and measured and analyzed the height, temperature and flow rate of the smoke layer below the smoke vent. In conclusion, in previous studies, CFD simulation method can better analyze the motion law of fluid [16–18].

The ventilation of the utility tunnel has been studied before. Curiel-Esparza and Canto-Perello [19] studied the ventilation of utility tunnel to reduce the harm of air pollution and provide a safe and healthy environment. Legrand et al. [20] proposed a multi standard method to make the utility tunnel safer and more sustainable, and studied the impact of the drainage network location on the total cost of the utility tunnel. However, there are some problems in the study of fire characteristics of cable compartment in utility tunnel. Firstly, the type of fire source in the cable compartment of utility tunnel is linear and square, and there are some differences in the temperature distribution in the cable compartment. Second, the location of the cable fire in the cable compartment of the utility tunnel may be close to one end of the shaft. Different longitudinal locations of fire sources have different effects on the development of cable indoor fire. Third, the constant temperature alarm device is used in the cable compartment of utility tunnel to reach the set alarm temperature, the transmission and exhaust port of the cable compartment of utility tunnel will be closed, so that the cable compartment forms a closed space. In this case, a special fire will occur in the cable compartment of utility tunnel. Therefore, it is of important significance to study the fire characteristics of fire smoke in the cable compartment of utility tunnel.

Therefore, this paper makes an experimental study on the maximum ceiling smoke temperature caused by cable indoor fire in the cable compartment of the utility tunnel. The effects of combustion time, oil pool, vertical height of fire source and ZRYJV combustion on combustion were studied. In fact, this fire scenario is more common in the cable compartment of utility tunnel. Through the small-scale cable compartment fire experiment, the smoke temperature distribution caused by fire under different HRR and cable types is simulated. These results are interpreted and compared and eventually used as validation data for the newly proposed empirical correlation of total HRR. This study provides some basic fire safety references for the utility tunnel scheme of urban underground cable compartment.

## 2. Method

### 2.1 Physical model and fire scenario

**Fig 1:**
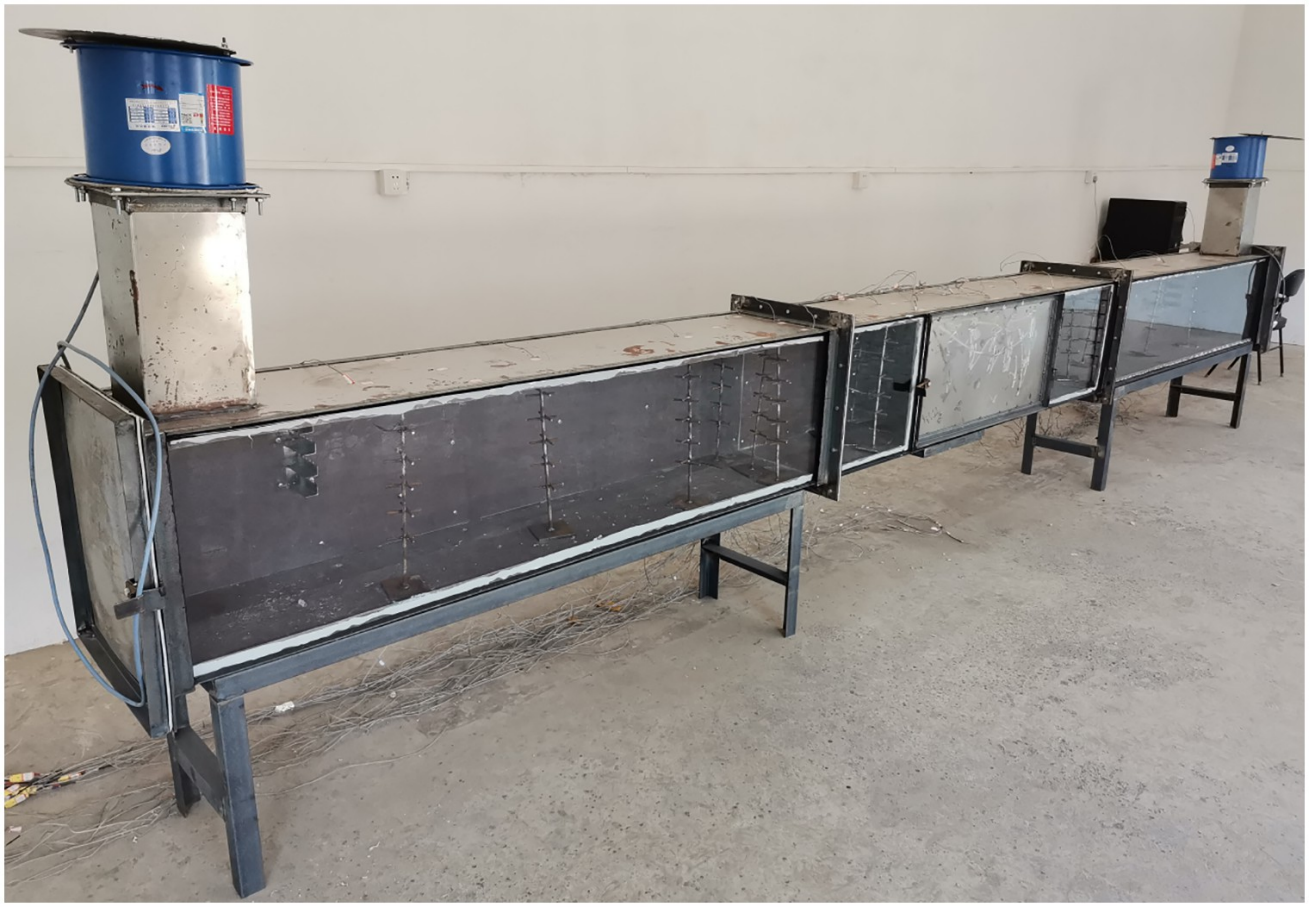
Physical model of cable compartment in utility tunnel.

According to the structural size of the cable compartment in utility tunnel in the actual project, a section of the length is selected as the research object. This length is sufficient to study the characteristics of fire smoke in a long and narrow space. The size of the cable compartment of utility tunnel selected is 36.0 m × 2.4 m × 3.0 m. In this paper, a small-scale model is used for fire experimental tests. The small-scale model size of the cable compartment is 6.0 m × 0.4 m × 0.5 m (Height × Width × High). The small-scale cable compartment is 0.5 m high from the ground. And it is supported by steel frame. The small scale of the model is 1:6. As shown in Fig 1, the physical model of the cable compartment in utility tunnel is established.

As shown in Table 1, the longitudinal ventilation status is showed in the cable compartment of utility tunnel. In this paper, nine cases of fire tests were carried out in the small-scale cable compartment of utility tunnel. Five experimental tests with fire source height of 0 m and 0.1 m are carried out without cables. For example, in cases 1 ~ 3 and in cases 8 ~ 9, the amount of fuel is different. Next, in the case of RVVR cable, the fire characteristics of different fuel quantities when the fire source height is 0 m are studied. In addition, when ZRYJV cable is used, the fire source characteristics of different fuel quantities are studied when the fire source height is 0.1 m. In this way, the orthogonal experiment method is used to study the influence characteristics of variables such as cable type, fuel quantity and fire source height. The fire source is 3.0 m away from the shaft of the utility tunnel. The fuel is n-heptane. Flame retardant crosslinked polyethylene sheathed PVC insulated cables are ZRYJV and RVVR cables. The flame retardant cable ZRYJV has 2 cores and an area of 10 mm^2^. The flame retardant cable RVVR has 1 cores and an area of 10 mm^2^. These two types of cables are typical and represent two commonly used cable materials. The natural ventilation velocity is 0 m/s.

**Table 1:** Cable tunnel fire simulation case.

Case	Fire source height (m)	Fuel quantity (ml)	Oil pool	Cable type	Cable length (m)
1	0	40	9.2×9.2	——	——
2	0	20	9.2×9.2	——	——
3	0	10	9.2×9.2	——	——
4	0	10	9.2×9.2	RVVR	0.6
5	0	20	9.2×9.2	RVVR	0.6
6	0.1	40	9.2×9.2	ZRYJV	0.6
7	0.1	20	9.2×9.2	ZRYJV	0.6
8	0.1	20	9.2×9.2	——	——
9	0.1	10	9.2×9.2	——	——

**Fig 2:**
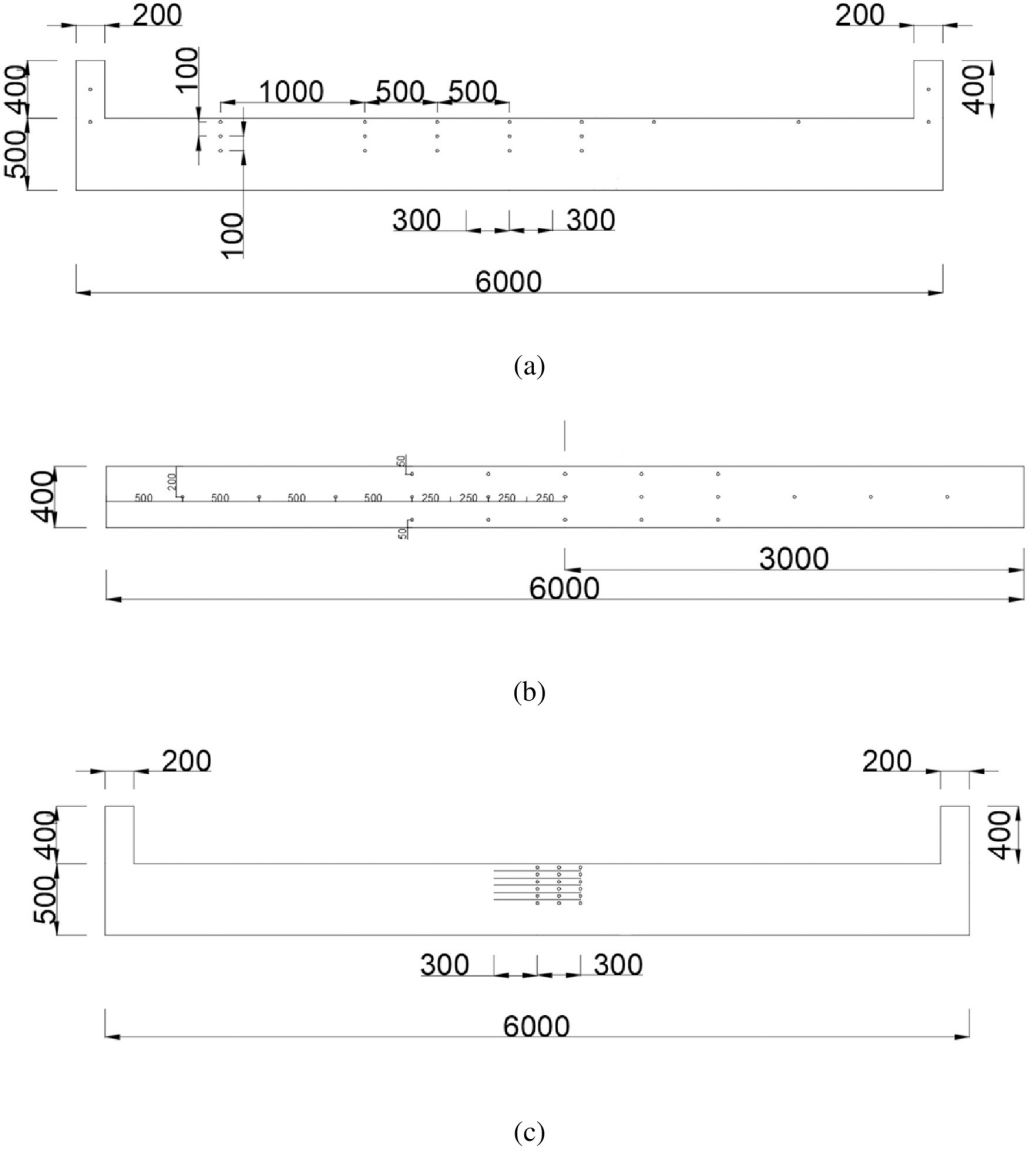
The layout of thermocouples.

**Fig 3:**
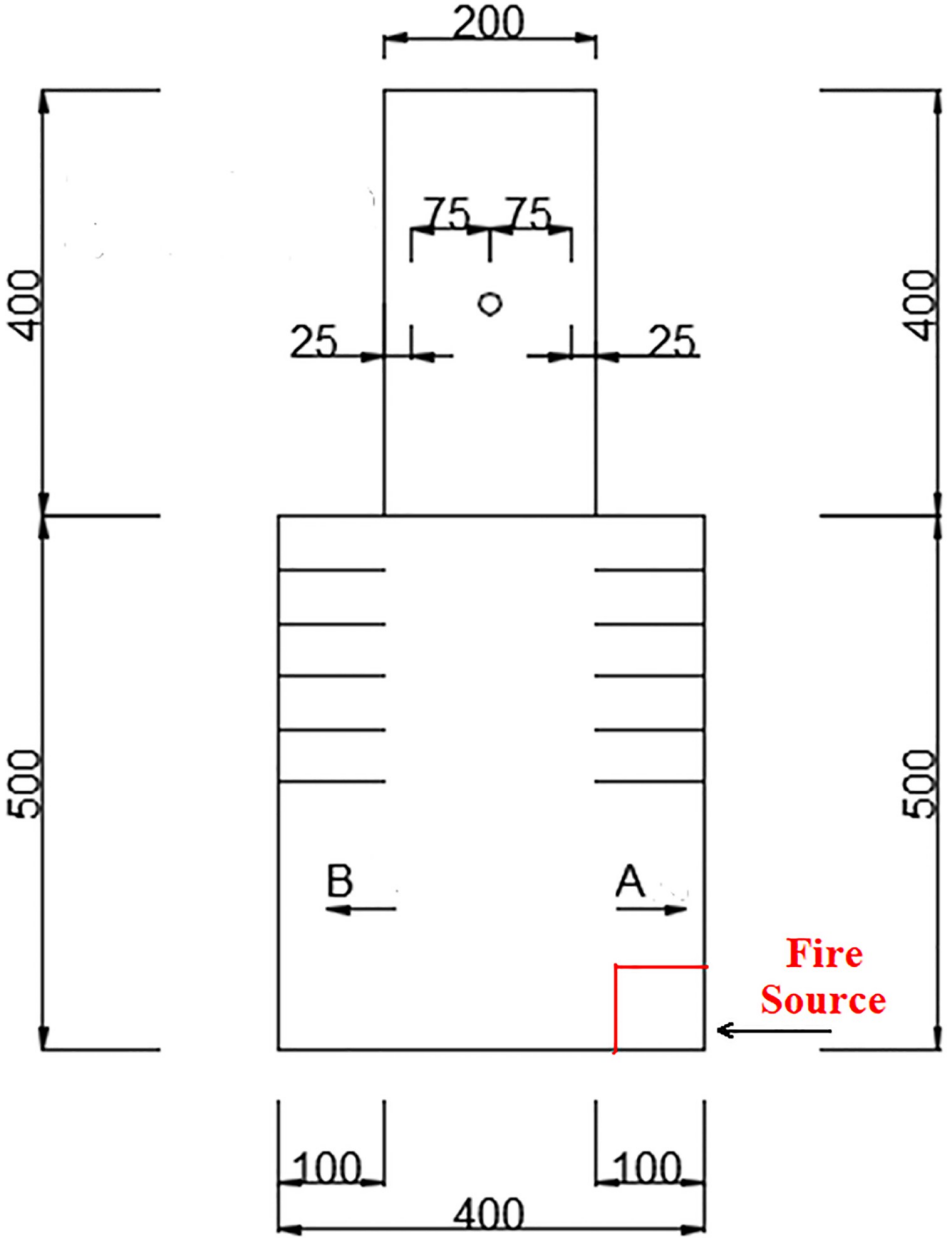
The schematic layout of fire source location of experimental cases.

As shown in Fig 2, the layout of thermocouple. For the thermocouple arrangement, 53 thermocouples are used. The thermocouple arrangement in the cable compartment of utility tunnel is as follows. Among them, 14 thermocouples are arranged on the longitudinal centerline of C, 21 thermocouples are arranged below the ceiling, and 18 thermocouples are arranged on one side. As shown in Fig 3, the schematic layout of fire source location of experimental cases.

In fact, in the case of natural ventilation, the experiments of each case were repeated three times. The experimental results of three times under each case are very close and have strong repeatability. The relative error is less than 5%. The experimental results meet the requirements of accuracy.

### 2.2 Theoretical analysis

In order to predict the total HRR of cable fire source, the total HRR measured in fire experimental test shall be calculated as follows:

$$\dot{Q}=Q_{burner}+Q_{cable}$$ (1)

where, *Q*_*burner*_ and *Q*_*cable*_ are ignition HRR and cable combustion HRR, respectively.

In this paper, we only consider the total HRR of fire source $\dot{Q}$, not the cable combustion HRR *Q*_*cable*_ and ignition source HRR *Q*_*burner*_ respectively. For the fire experimental tests cases near the side wall, it is used Zukosi [21] mirror model to express the total HRR as shown in the Eq (2). By measuring and recording the temperature below the ceiling of the utility tunnel, the total HRR of the fire source is calculated by Eq (2).

$$\dot{Q}={\left[\frac{\Delta T_{dp}}{4.73\times \vartheta \times T_{\infty }}{\left(c_{p}\rho T\sqrt{g}\right)}^{2/3}H_{ef}^{5/3}{\left(0.6+\frac{r}{H}\right)}^{4/3}\right]}^{3/2}$$ (2)

where, *ϑ* = 1.59, Δ*T*_*dp*_ is temperature below the ceiling, *T*_∞_ is ambient temperature, *c*_*p*_ is constant pressure specific heat capacity, *ρ* is smoke density, *g* is gravitational acceleration, *H*_*ef*_ is effective height of fire source, *r* is fire plume radius, *H* is height of utility tunnel.

In the fire research of cable compartment of utility tunnel, the method of introducing correction coefficient is adopted to calculate the fire source HRR and establish the model of fire HRR, as shown in Eq (3).

$$\dot{Q}={\left[\frac{\Delta T_{dp}}{\vartheta \times T_{\infty }}{\left(c_{p}\rho T\sqrt{g}\right)}^{2/3}H_{ef}^{5/3}{\left(0.6+\frac{r}{H}\right)}^{4/3}\right]}^{3/2}$$ (3)

where, *ϑ* = 1.567.

## 3. Results and discussion

### 3.1 Combustion time

In this paper, the total combustion time caused by cable fire in the cable compartment of the utility tunnel is experimentally studied. As shown in Table 2, the combustion of cables and the total duration of fuel combustion can be seen from the combustion time of fire in the cable compartment. ZRYJV cable burns more fully than RVVR cable. When the cable type is ZRYJV, the more cable layers, the more sufficient combustion, the longer combustion duration, and the greater cable quality loss. The time when the cable is ignited is closely related to the experimental conditions. The time when the cable is ignited is different in each case.

**Table 2:** Cable tunnel fire simulation case.

Case	Cable	Number of cable layers	Is the cable ignited	Cable burning loss mass (g)	Total duration of combustion (s)
1	——				382
2	——				213
3	——				121
4	RVVR	2	NO	0	125
5	RVVR	2	Yes	13	251
6	ZRYJV	2	Yes	53	689
7	ZRYJV	4	Yes	338.5	925
8	——				342
9	——				144

### 3.2 Influence of oil pool type

**Fig 4:**
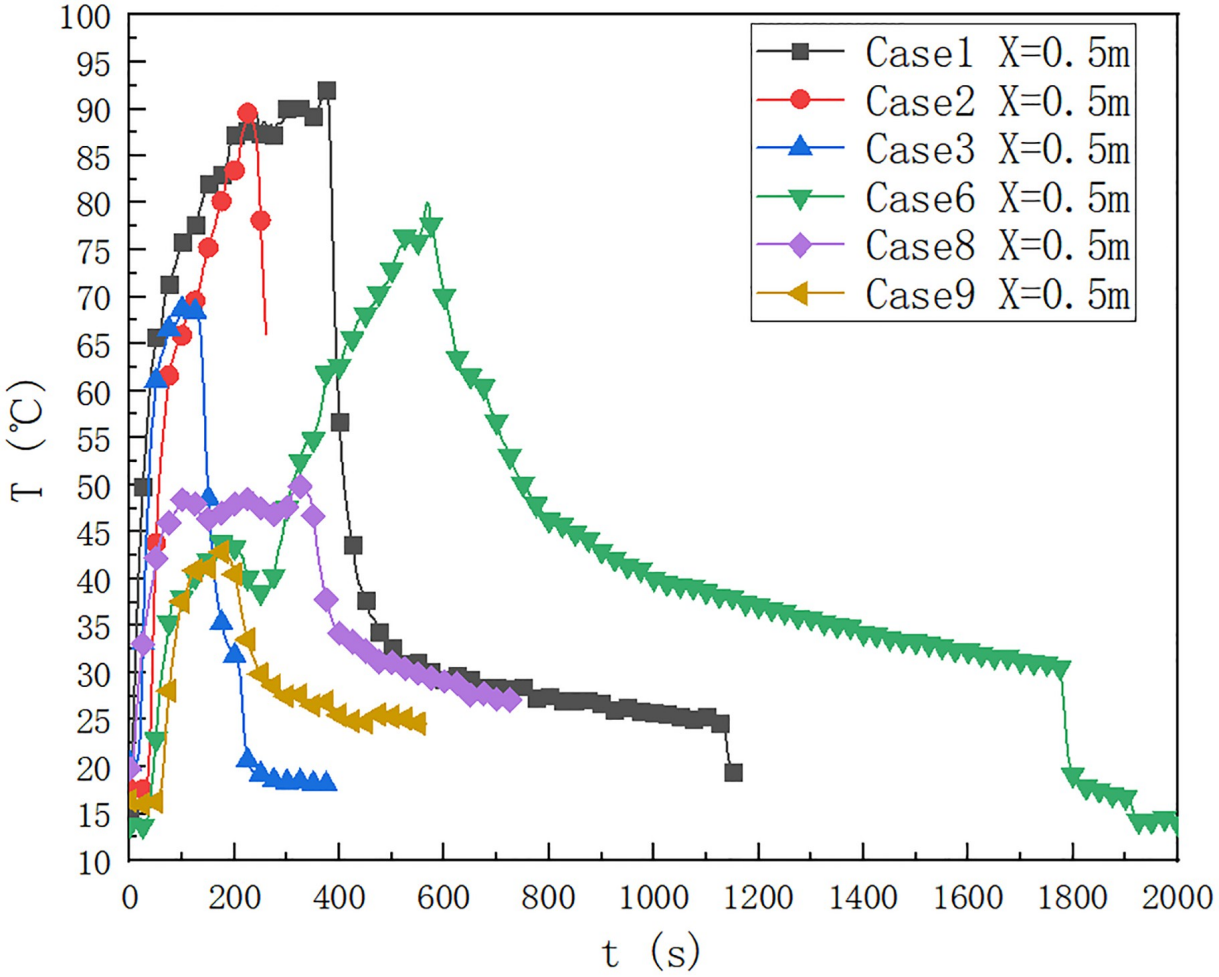
Temperature change below the ceiling of the cable compartment in the utility tunnel at 1, 2, 3, 6, 8 and 9 at a distance of 0.5 m from the fire source.

As shown in Fig 4, the type of oil pool is 9.2×9.2 cm, in the cases of 1, 2, 3, 6, 8 and 9, the temperature change below the ceiling of the cable compartment 0.5 m away from the fire source. It can be seen that when the fuel quantity is 40 ml, the upper limit temperature reaches 90°C for a long time, and the fuel quantity affects the duration when the maximum temperature is lower than the upper limit. When the fuel quantity is 10 ml, the maximum temperature below the ceiling is 70°C, which is lower than the maximum temperature below the ceiling when the fuel quantity is 20 ml. When the height of the fire source is 0.1 m, the temperature distributions of case 6, case 8 and case 9 are compared, when the fuel quantity is 40 ml, 20 ml and 10 ml respectively. It can be seen that when the fuel quantity is 40 ml, the upper limit temperature reaches 80°C for a long time, and the fuel quantity affects the maximum temperature 0.5 m below the ceiling. When the fuel quantity is 10 ml, the maximum temperature below the ceiling is about 45°C, which is lower than 47°C when the fuel quantity is 20 ml.

**Fig 5:**
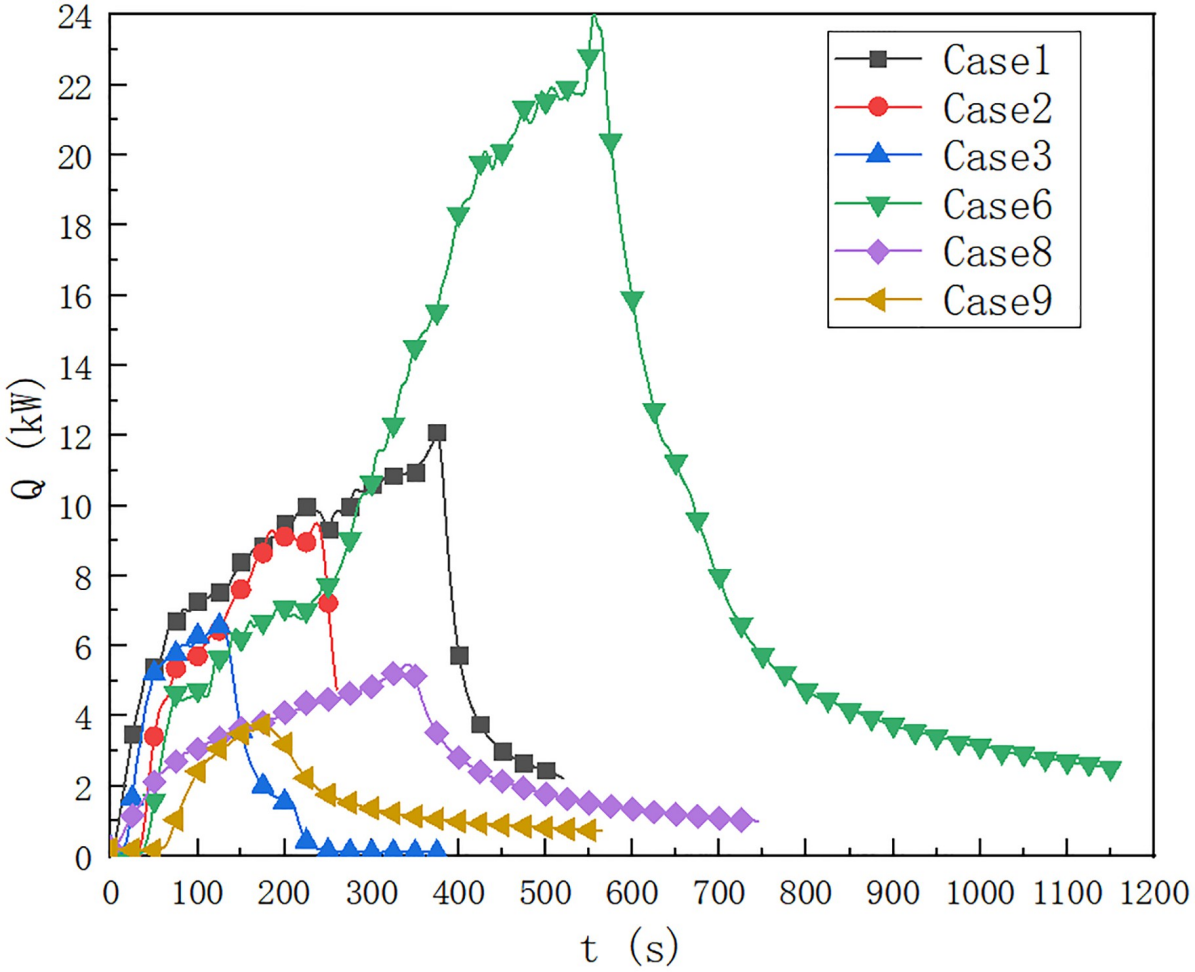
Prediction of fire HRR at 0.5 m from fire source in cable compartment of utility tunnel in cases of 1, 2, 3, 6, 8 and 9.

As shown in Fig 5, in case of 1, 2, 3, 6, 8 and 9, the oil pool type is 9.2×9.2 cm, HRR is predicted by the temperature below the ceiling of the cable compartment 0.5 m away from the fire source. When the height of the fire source is 0 m, the fuel quantity is 40 ml, 20 ml and 10 ml respectively, compared with the temperature distribution of case 1, case 2 and case 3. It can be seen that when the fuel quantity is 40 ml, the maximum HRR of the fire source reaches 12 kW, and the fuel quantity affects the HRR of the fire source. When the fuel quantity is 10 ml, the maximum HRR of the fire source is 6.3 kW, which is lower than 9.2 kW when the fuel quantity is 20 ml.

In cases 6, 8 and 9, it can be seen that when the fuel quantity is 40 ml, the upper limit temperature reaches 24 kW for a long time. When the fuel quantity is 10 ml, the HRR is about 3 kW, which is lower than the upper limit temperature below the upper limit of 5 kW when the fuel quantity is 20 ml.

**Fig 6:**
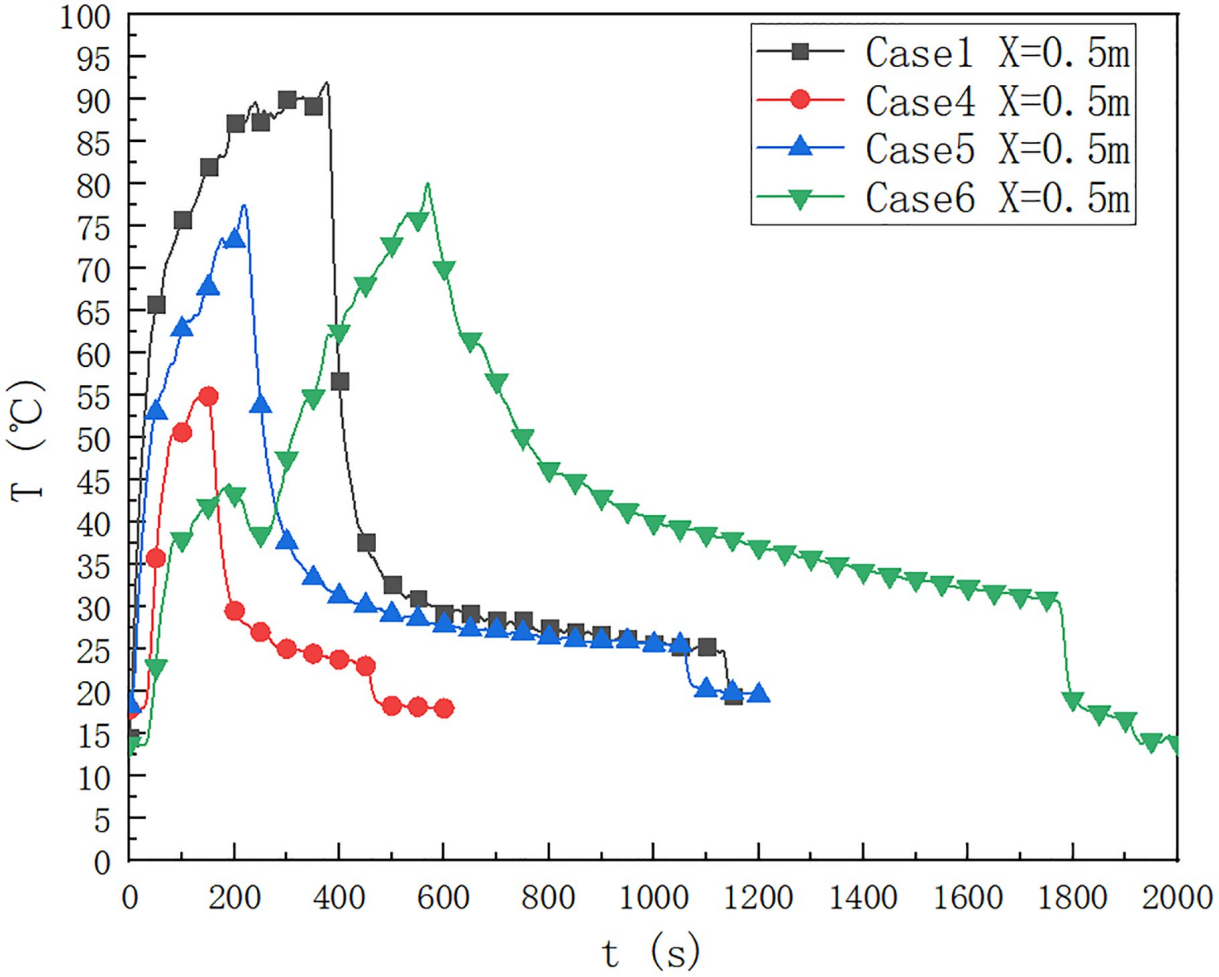
Temperature change below the ceiling of the cable compartment in the utility tunnel at 1, 4, 5 and 6 at a distance of 0.5 m from the fire source.

As shown in Fig 6, the square oil pool is 9.2×9.2 cm, in the case of 1, 4, 5 and 6, the temperature change below the ceiling of the cable compartment is 0.5 m away from the fire source. It can be seen that when the fuel quantity is 40 ml, the duration of the maximum temperature reaching 90°C is relatively long, and the fuel quantity affects the duration for which the temperature below the ceiling reaches the maximum. When the fuel quantity is 10 ml, the RVVR cable is not ignited; When the fuel quantity is 20 ml, the RVVR cable is ignited. When the fuel quantity is 10 ml, the maximum temperature below the ceiling is about 55°C, which is lower than 75°C when the fuel quantity is 20 ml.

In cases 1, 5 and 6, it can be seen that when the fuel quantity is 40 ml, in case 1 and 6 without cable, the maximum ceiling temperature reaches 90°C in a relatively long time, and the fuel quantity affects the maximum temperature, which is 0.5 m below the ceiling. When the fuel quantity is 10 ml, the maximum temperature below the ceiling of RVVR ignition cable is about 74°C, which is lower than the maximum temperature 77°C below the ceiling of ZRYJV ignition cable 0.5 m away from the fire source when the fuel quantity is 40 ml.

**Fig 7:**
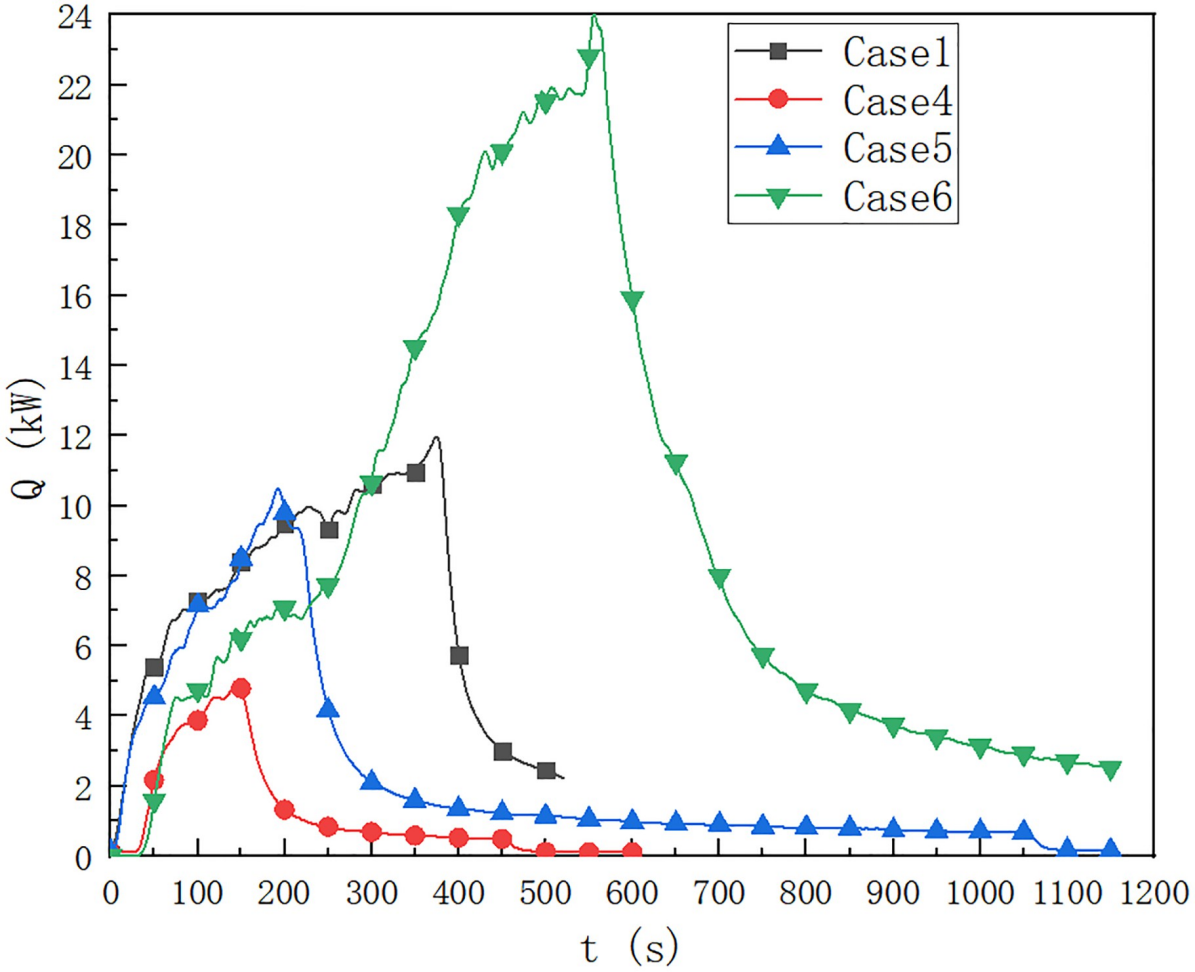
Prediction of fire HRR at 0.5 m from fire source in cable compartment of utility tunnel in cases of 1,4,5 and 6.

As shown in Fig 7, in cases 1, 4 and 5, it can be seen that when the fuel quantity is 40 ml, the maximum HRR of fire source reaches 12 kW, and the fuel quantity affects the HRR of the fire source. When the fuel quantity is 10 ml, the maximum HRR of the ignition source is about 5.0 kW, which is lower than 10.3 kW when the fuel quantity is 20 ml.

In cases 1, 5 and 6, it can be seen that when the fuel quantity is 40 ml, the ignition HRR of cable type ZRYJV reaches 24 kW and lasts for a long time. The fuel quantity affects the HRR of the fire source. When the fuel quantity is 20 ml, the ignition HRR of cable type RVVR is about 10.3 kW, which is lower than that of cable ZRYJV when the fuel quantity is 40 ml, which is 11.5 kW.

### 3.3 Influence of vertical height of fire source

**Fig 8:**
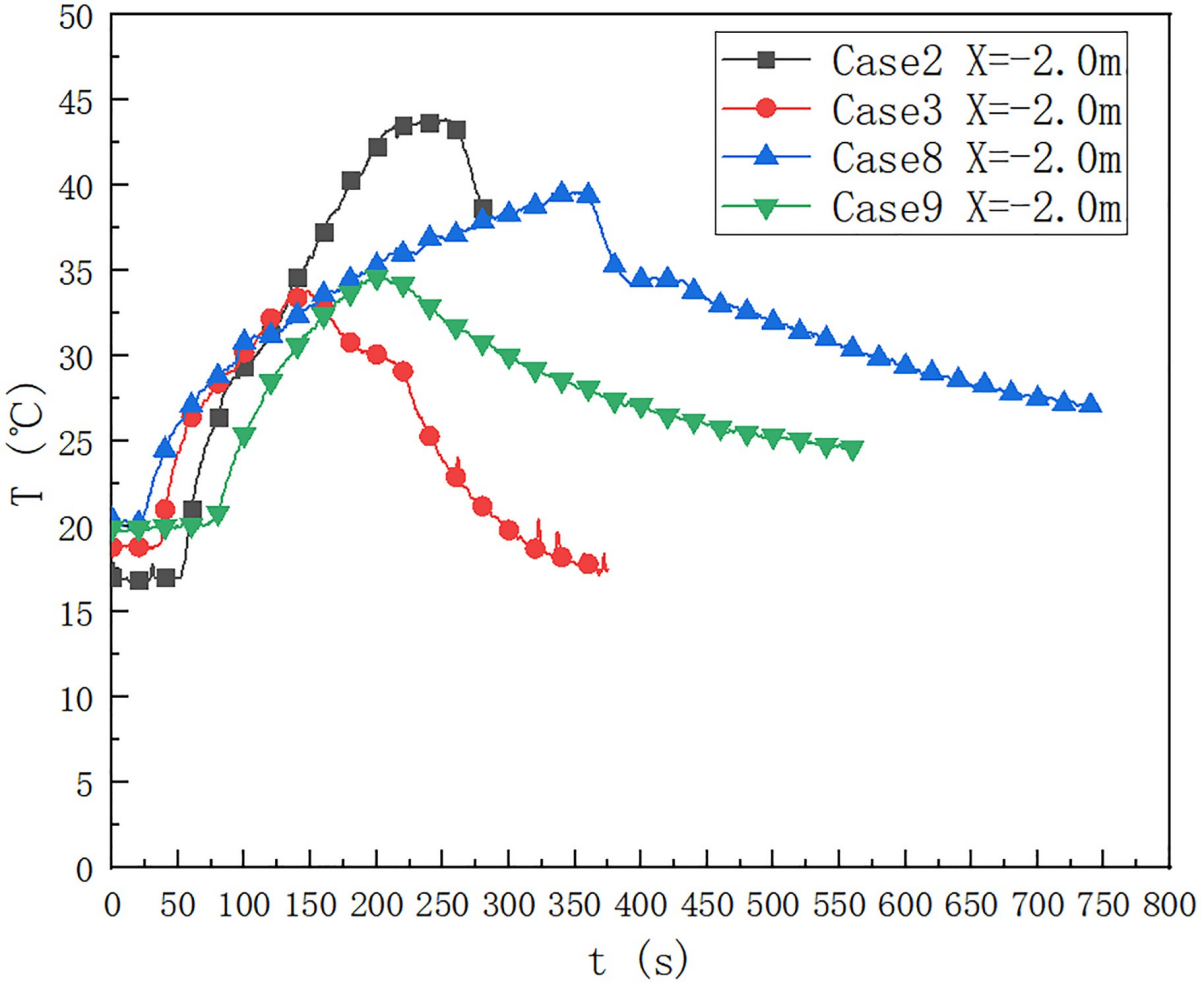
Temperature change below the ceiling of the cable compartment in the utility tunnel at 2,3,8 and 9 at a distance of -2.0 m from the fire source.

As shown in Fig 8, the square oil pool is 9.2×9.2 cm, in cases 2, 3, 8 and 9, the temperature change below the ceiling of the cable compartment 2.0 m upstream from the fire source. It can be seen that when the fuel quantity is 20 ml, the maximum temperature 2.0 m upstream from the fire source lasts for a long time. And the fuel quantity affects the duration for which the maximum temperature below the ceiling. When the fuel quantity is 20 ml, the fire source height is 0 m in case 2. When the fuel quantity is 20 ml, the fire source height is 0.1 m. When the fire source height is 0 m, the maximum temperature below the ceiling 2.0 m upstream of from the fire source is 43.9°C, which is higher than the maximum temperature below the ceiling 0.1 m. When the fire source height is 0 m and the fire source height is 0.1 m, the maximum temperature below the ceiling is 148 s earlier than 337 s.

When the height of the fire source is 0 m, the maximum temperature below the ceiling 2.0 m upstream of the fire source is about 34°C, which is higher than the maximum temperature 0.1 m below the ceiling. When the fire source height is 0 m, the maximum temperature below the ceiling reaches 148 s, which is earlier than 205s when the fire source height is 0.1 m, and the maximum temperature below the ceiling reaches 34.6°C.

**Fig 9:**
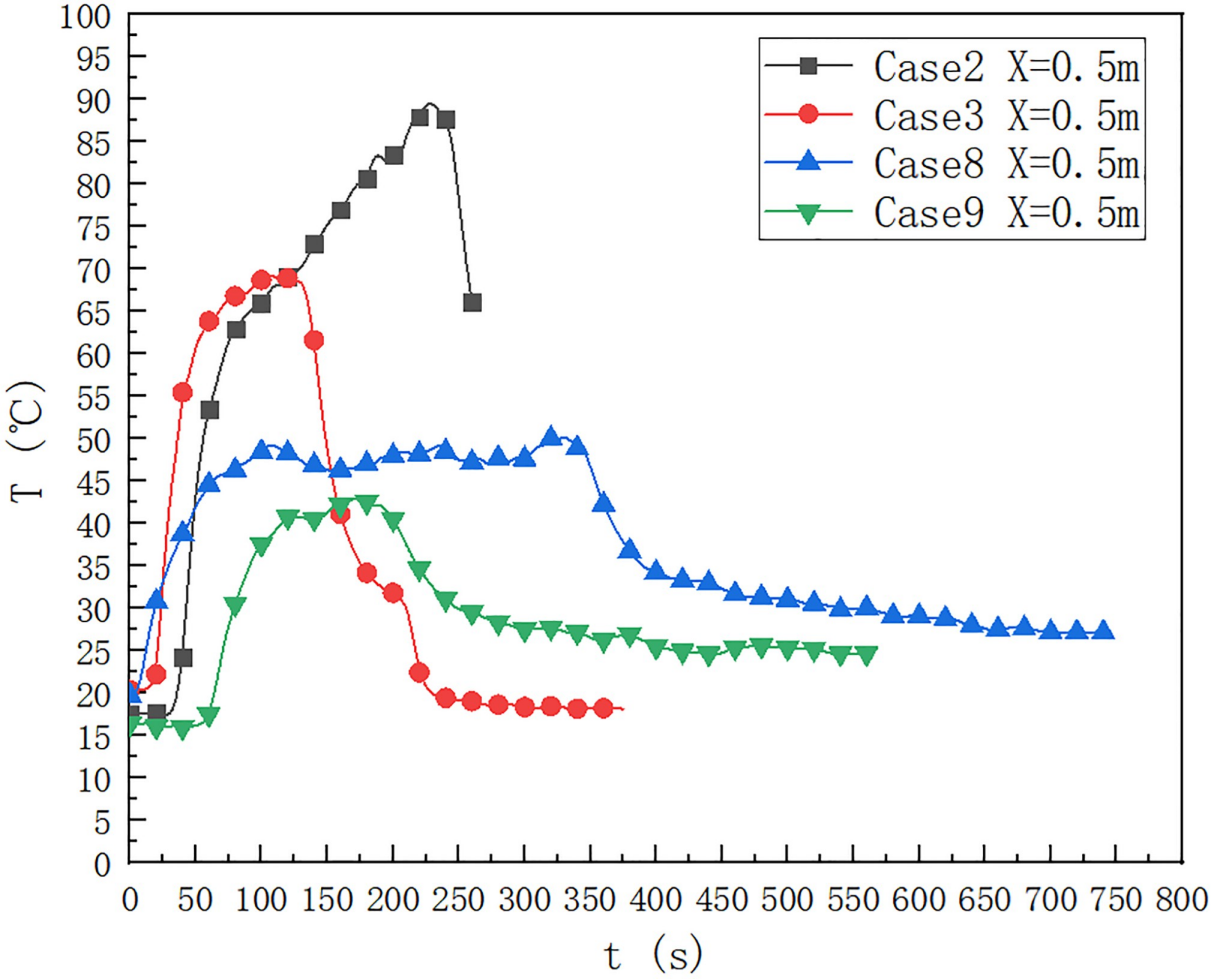
Temperature change below the ceiling of the cable compartment in the utility tunnel at 2,3,8 and 9 at a distance of 0.5 m from the fire source.

As shown in Fig 9, in cases 2, 3, 8 and 9, the temperature below the ceiling of the cable compartment 2.0 m upstream of the fire source. It can be seen that when the fuel quantity is 20ml, the ceiling temperature at 0.5 m away from the fire source is about 89.55°C for a long time, and the fuel quantity affects the duration of the maximum temperature below the ceiling. When the fuel quantity is 20 ml, the fire source height is 0 m in case 2. When the fuel quantity is 20 ml, the fire source height is 0.1 m. When the fire source height is 0 m, the maximum temperature below the ceiling 0.5 m away from the fire source is 89.55°C, which is higher than 50.0°C when the fire source height is 0.1 m. When the fire source height is 0 m, the maximum temperature below the ceiling is 225 s earlier than 320 s when the fire source height is 0.1 m.

In cases 3 and 9. When the height of the fire source is 0 m, the maximum temperature below the ceiling 0.5 m away from the fire source is 69.2°C, which is higher than 42.9°C when the height of the fire source is 0.1 m. When the fire source height is 0 m, the maximum temperature below the ceiling reaches 69.2°C and the duration is 110 s. When the fire source height is 0.1 m, the maximum temperature below the ceiling reaches 42.9°C and the duration is 170 s.

**Fig 10:**
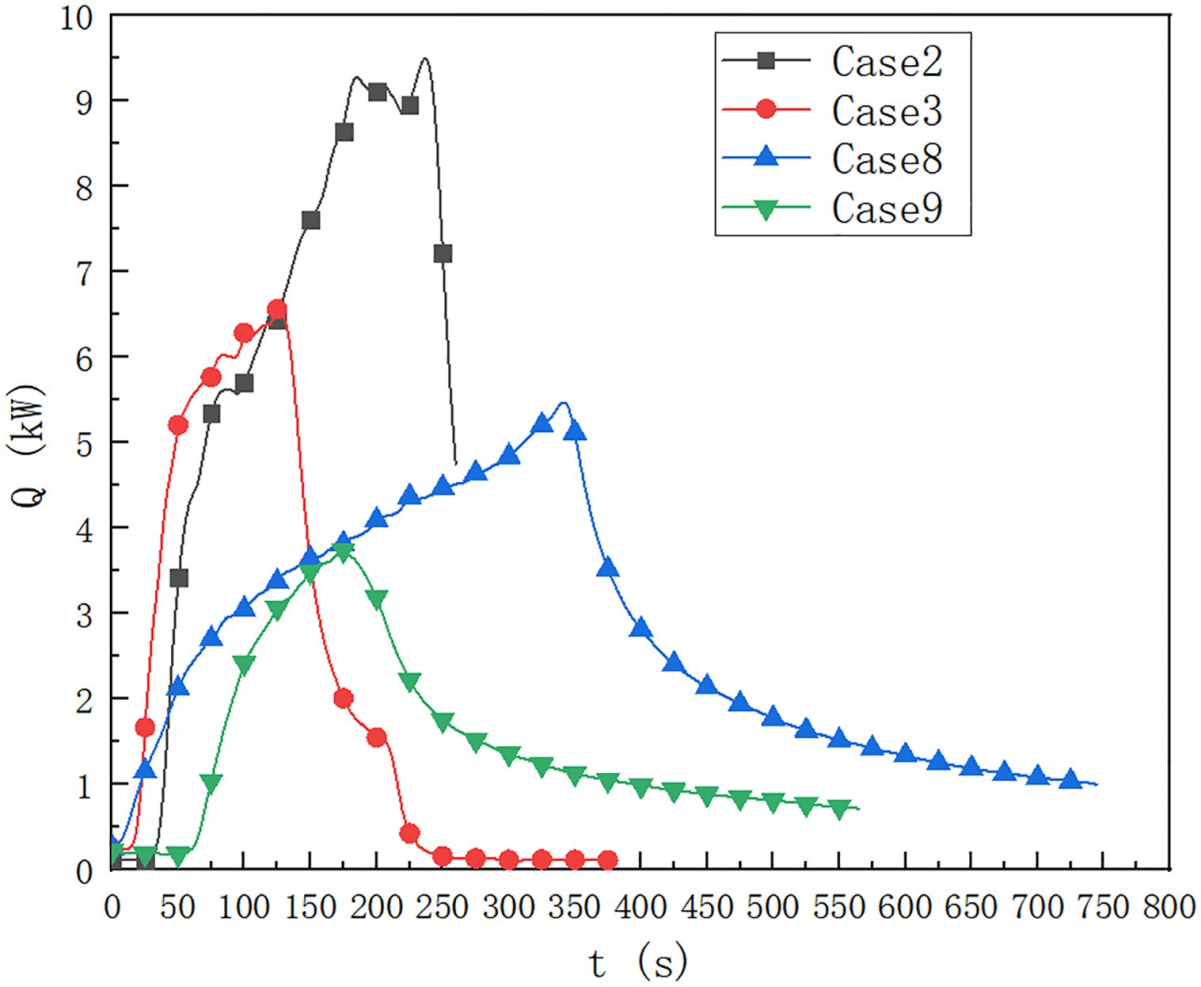
Prediction of fire HRR at 0.5 m from fire source in cable compartment of utility tunnel under cases of 2, 3, 8 and 9.

As shown in Fig 10, the square oil pool is 9.2×9.2 cm. In cases 2, 3, 8 and 9, the fire source HRR is predicted according to the ceiling and bottom temperature prediction at 0.5 m away from the fire source in the cable compartment of utility tunnel. It can be seen that when the fuel quantity is 20ml, the HRR is significantly higher than 10 ml fuel, and the fuel quantity affects the HRR. When the fuel quantity is 20 ml, the fire source height is 0 m in case 2. When the fuel quantity is 20 ml, the fire source height is 0.1 m. When the fire source height is 0 m, the maximum HRR of the fire source is 9.24 kW, and when the height of the fire source is 0.1 m, the maximum HRR of the fire source is 5.49 kW. When the height of fire source is 0 m, the time required for HRR to reach the maximum value is 205 s. When the height of fire source is 0.1 m, the time required for HRR to reach the maximum value is 340 s.

In cases 3 and 9, when the fire source height is 0 m, the maximum fire source HRR is about 6.56 kW, which is higher than that when the fire source height is 0.1 m, the maximum fire source HRR is 3.74 kW. When the fire source height is 0 m, the time required for the fire source HRR to reach the maximum value is 125 s, which is earlier than the time required for the fire source HRR to reach the maximum value of 3.74 kW when the fire source height is 0.1 m.

### 3.4 Influence of cable type ZRYJV

**Fig 11:**
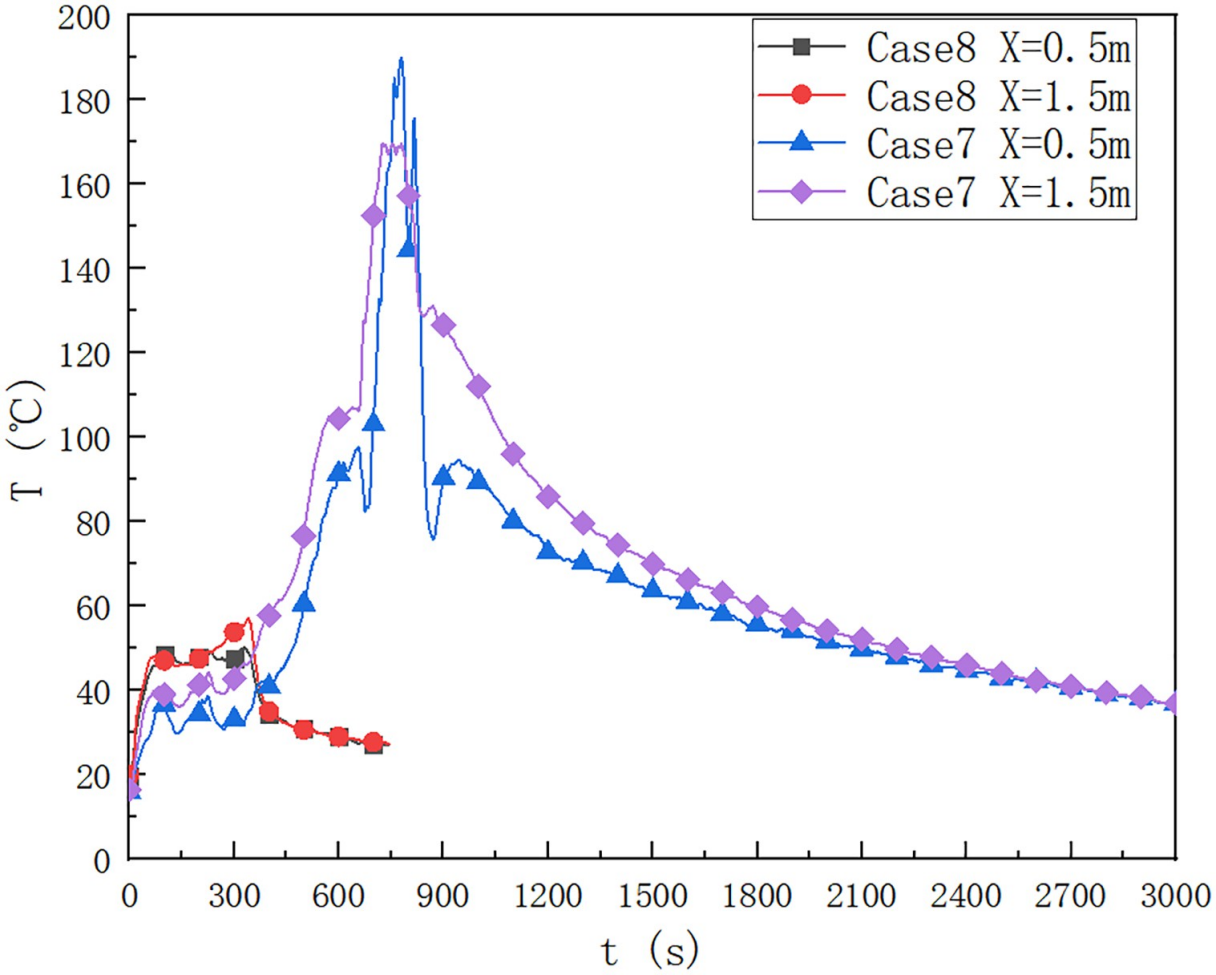
Temperature below the ceiling of the cable compartment in the utility tunnel at 7 and 8 at a distance of 0.5 m and 1.5 m from the fire source.

As shown in Fig 11, in cases 7 and 8, the temperature change below the ceiling in the cable compartment of utility tunnel is 0.5 m and 1.5 m away from the fire source, respectively. When the cable type ZRYJV is placed, the comparison between case 7 and case 8 shows that when the cable type ZRYJV is placed in case 7, the time required for the ceiling temperature at 0.5 m away from the fire source to reach 190.5°C is 780 s, and the time required for the maximum temperature at 1.5 m away from the fire source to reach 170.5°C is 755 s. When the cable is not laid, the time required for the ceiling temperature to reach 50.0°C at a distance of 0.5 m from the fire source is 320 s, and the time required for the ceiling temperature to reach 57.4°C at a distance of 1.5 m from the fire source is 340 s. It takes a long time to place the ZRYJV cable to reach the maximum temperature below the ceiling.

**Fig 12:**
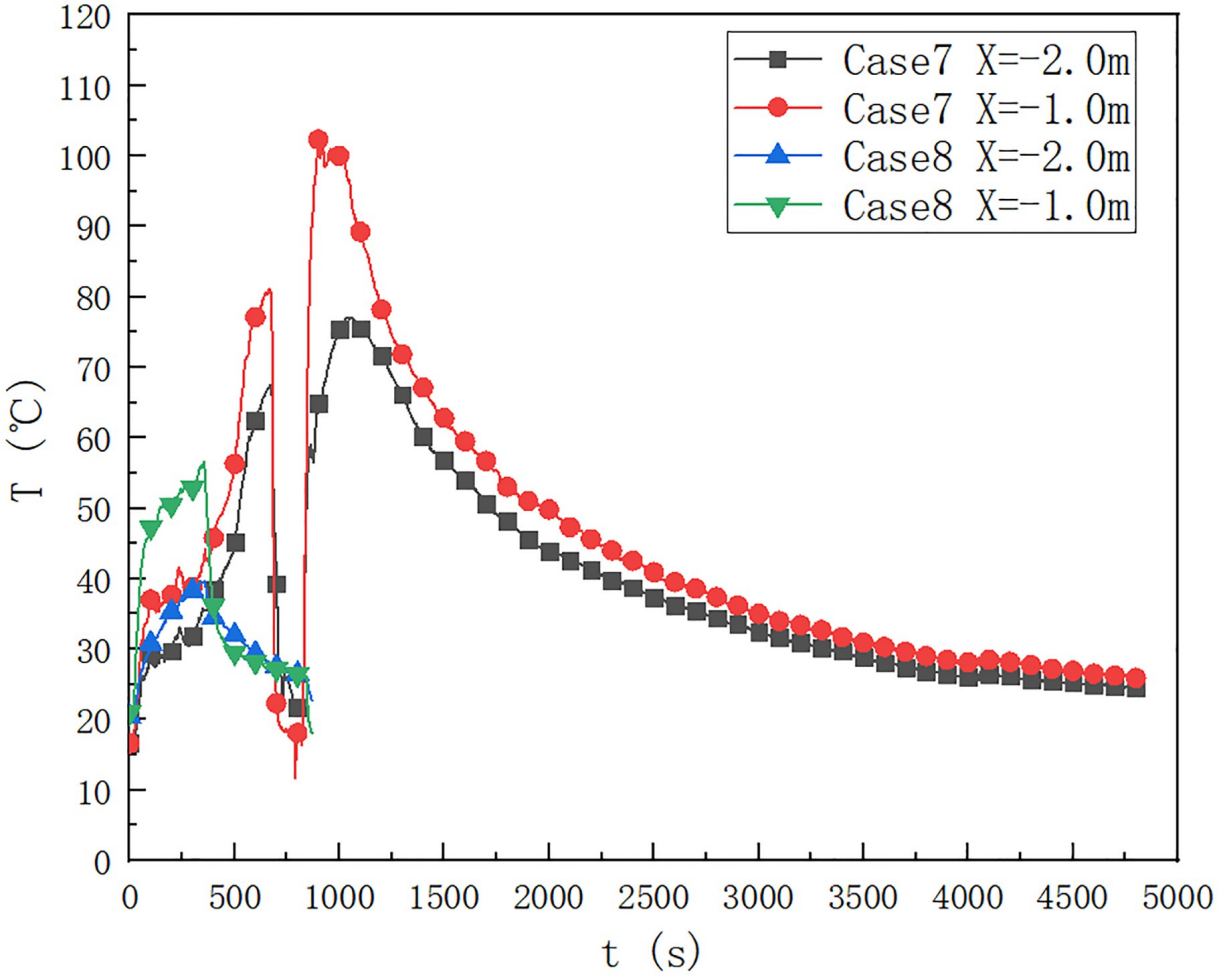
Temperature below the ceiling of the cable compartment in utility tunnel at 7 and 8 at a distance of -1.0 m and -2.0 m from the fire source.

As shown in Fig 12, in cases 7 and 8, the temperature change below the ceiling in the cable compartment of utility tunnel is 1.0 m and 2.0 m upstream of the fire source. When placing ZRYJV cable, it can be seen that in case 7, the ceiling temperature at 2.0 m upstream of the fire source is about 67.5°C, and the ceiling temperature at 1.0 m upstream of the fire source is about 80.9°C for 670 s. When the cable is not laid, the ceiling temperature at 2.0 m upstream of the fire source is about 39.7°C for 335 s. And the time required for the ceiling temperature at 1.0 m upstream of the fire source is about 350s. Placing ZRYJV cable will affect the time required to reach the maximum temperature below the ceiling.

In this paper, the temperature and HRR of cable compartment of the utility tunnel are studied. Two factors are considered, namely, the vertical height of the fire source and the type of cable. Through experimental research, the fire characteristics of fire source set at 3.0 m of the air supply shaft in the cable compartment of the utility tunnel are studied. These results are interpreted and compared with each other, and finally used as validation data for the newly proposed empirical correlation of total HRR, as shown in Eq (3).

## 4. Conclusions

In this paper, the maximum smoke temperature of ceiling caused by fire under the condition of natural ventilation in the cable compartment of utility tunnel is experimentally studied. The fire source of the oil pool is located on one side of the cable compartment of the utility tunnel, forming a fire near the wall. The effects of combustion time, oil pool, vertical height of fire source and ZRYJV cable combustion on combustion are studied. In fact, this fire scenario is more common in the cable compartment of utility tunnel. Through the fire model test of the cable compartment in the utility tunnel, the smoke temperature distribution caused by fire under different HRR and cable types is simulated.

The results showed that under the action of natural ventilation, with the decrease of fuel quantity, the mass loss of cable decreases, and the maximum temperature below the ceiling of the cable compartment in the utility tunnel decreases. ZRYJV cable burns more fully than RVVR cable. A new empirical correlation of total HRR is proposed. These experimental test results are used as validation data for the newly proposed empirical correlation of total HRR. This study provides some basic fire safety references for the utility tunnel scheme of urban underground cable compartment.
